# Visual impairment and retinal and brain neurodegeneration: A population‐based study

**DOI:** 10.1002/hbm.26237

**Published:** 2023-02-28

**Authors:** Davide Garzone, Robert P. Finger, Matthias M. Mauschitz, Alexandra Koch, Martin Reuter, Monique M. B. Breteler, N. Ahmad Aziz

**Affiliations:** ^1^ Population Health Sciences German Center for Neurodegenerative Diseases (DZNE) Bonn Germany; ^2^ Department of Ophthalmology, Faculty of Medicine University of Bonn Bonn Germany; ^3^ Image Analysis German Center for Neurodegenerative Diseases (DZNE) Bonn Germany; ^4^ A.A. Martinos Center for Biomedical Imaging Massachusetts General Hospital Boston Massachusetts USA; ^5^ Department of Radiology Harvard Medical School Boston Massachusetts USA; ^6^ Faculty of Medicine Institute for Medical Biometry, Informatics and Epidemiology (IMBIE), University of Bonn Bonn Germany; ^7^ Department of Neurology, Faculty of Medicine University of Bonn Bonn Germany

**Keywords:** brain, modeling, neurodegeneration, retina, structural equation, visual impairment

## Abstract

Visual impairment and retinal neurodegeneration are intrinsically connected and both have been associated with cognitive impairment and brain atrophy, but the underlying mechanisms remain unclear. To investigate whether transneuronal degeneration is implicated, we systematically assessed the relation between visual function and retinal, visual pathway, hippocampal and brain degeneration. We analyzed baseline data from 3316 eligible Rhineland Study participants with visual acuity (VA), optical coherence tomography (OCT), and magnetic resonance imaging (MRI) data available. Regional volumes, cortical volume, and fractional anisotropy (FA) were derived from T1‐weighted and diffusion‐weighted 3 T MRI scans. Statistical analyses were performed using multivariable linear regression and structural equation modeling. VA and ganglion cell layer (GCL) thinning were both associated with global brain atrophy (SD effect size [95% CI] −0.090 [−0.118 to −0.062] and 0.066 [0.053–0.080], respectively), and hippocampal atrophy (−0.029 [−0.055 to −0.003] and 0.114 [0.087–0.141], respectively). The effect of VA on whole brain and hippocampal volume was partly mediated by retinal neurodegeneration. Similarly, the effect of retinal neurodegeneration on brain and hippocampal atrophy was mediated through intermediate visual tracts, accounting for 5.2%–23.9% of the effect. Visual impairment and retinal neurodegeneration were robustly associated with worse brain atrophy, FA, and hippocampal atrophy, partly mediated through disintegration of intermediate visual tracts. Our findings support the use of OCT‐derived retinal measures as markers of neurodegeneration, and indicate that both general and transneuronal neurodegeneration along the visual pathway, partly reflecting visual impairment, account for the association between retinal neurodegeneration and brain atrophy.

## Graphical abstract

Visual impairment and retinal neurodegeneration are associated with brain atrophy and brain integrity in areas involved in visual processing and cognition.

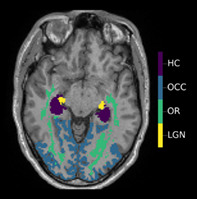

## 1 OBJECTIVES

The retina is the first relay of the visual pathway, responsible for converting light signals into axon potentials. Retinal pathology has consistently been associated with degeneration of various components of the visual pathway, including the primary visual area (V1), the optic radiation, and the lateral geniculate nucleus (Boucard et al., 2009; Hernowo et al., 2011, 2014; Malania et al., 2017; Mutlu et al., 2018; Prins et al., 2016). This could be due to transneuronal degeneration, since these structures are components of the geniculostriate pathway: axons of the retinal ganglion cells (GCL) bundle together in the retinal nerve fiber layer (RNFL), and subsequently synapse at the level of the lateral geniculate nucleus, conveying visual information to V1 through the optic radiation. From V1, visual information is then carried forward through the ventral (parietal) and dorsal (temporal) streams for further integration and multimodal processing (Mishkin et al., 1983). In particular, retinal thinning in the inner neuronal layers (including GCL, the inner plexiform layer [IPL], and RNFL), which can be assessed noninvasively with spectral domain—optical coherence tomography (SD‐OCT), is attributed to neurodegeneration and likely reflects neuronal damage accrued with age and other pathologies (Garzone et al., 2022; Mauschitz et al., 2018; Pfeiffer et al., 2020). Indeed, retinal neurodegeneration has been associated with both global and regional brain atrophy, as well as cognitive impairment (Hernowo et al., 2011, 2014; Ward et al., 2020; Mutlu et al., 2017).

Retinal neurodegeneration is intrinsically linked to visual function. For example, in glaucoma inner retinal neurodegeneration is the key event leading to visual loss, but an association between retinal neurodegeneration and visual loss is also seen in other retinal pathologies without primary inner retinal involvement. (Abdolrahimzadeh et al., 2019; Chiang et al., 2020; Li et al., 2021; Pillay et al., 2018; van Dijk et al., 2011) Decreased visual stimulation might also induce retinal degeneration through reduced levels of neurotrophic factors (Fleitas et al., 2020). Interestingly, visual impairment has also been associated with a higher risk of cognitive impairment (Chen et al., 2017; Shang et al., 2021, Ward et al., 2020), which is not surprising given that up to half of the total cortical surface area is devoted to the processing of visual information (Zilles & Amunts, 2012). Recently, it was shown that retinal neurodegeneration is preferentially associated with changes in the visual pathway, including the occipital lobe gray matter (Mutlu et al., 2018), and the medial temporal lobe structures, including the hippocampus (Kravitz et al., 2011). Indeed, temporal lobe structures are crucial for higher order visuospatial integration of downstream signals originating from the occipital lobe visual areas (Silson et al., 2020; Zilles & Amunts, 2012). Visual impairment and retinal pathology have also been associated with altered connectivity between visual and other brain areas (Sanda et al., 2018; Sabbah et al., 2017; Mendola et al., 2018; Huang et al., 2016; Collignon et al., 2013). However, to the best of our knowledge, systematic studies assessing the conjoint relations among visual impairment, retinal layer measures, major brain regions of the central visual system and involved in cognition and neurodegeneration, such as whole brain and hippocampal volume, in the general population are still lacking.

Accumulating evidence indicates that multisensory stimulation may enhance brain plasticity, and ward off neurodegeneration (Yang et al., 2021). In particular, visual stimulation has been demonstrated to decrease Alzheimer disease‐associated pathology and improve cognition in various mouse models (Iaccarino et al., 2016; Martorell et al., 2019). Nevertheless, the major underlying neuroanatomical pathways through which visual stimulation or impairment could affect brain structure and connectivity in humans remain elusive. Elucidating the pathways underlying the association between visual impairment, retinal neurodegeneration, and brain atrophy in the general population is of paramount importance for the development of more effective, targeted preventive and therapeutic paradigms against age‐associated neurodegeneration and cognitive decline. Therefore, using a population‐based approach, in this study, we aimed to systematically assess (1) the interrelations among visual impairment, retinal degeneration, and regional and general brain atrophy, and (2) whether transneuronal degeneration along the visual pathway could be implicated.

## 2 MATERIALS AND METHODS

### 2.1 Study design and population

**FIGURE 1:**
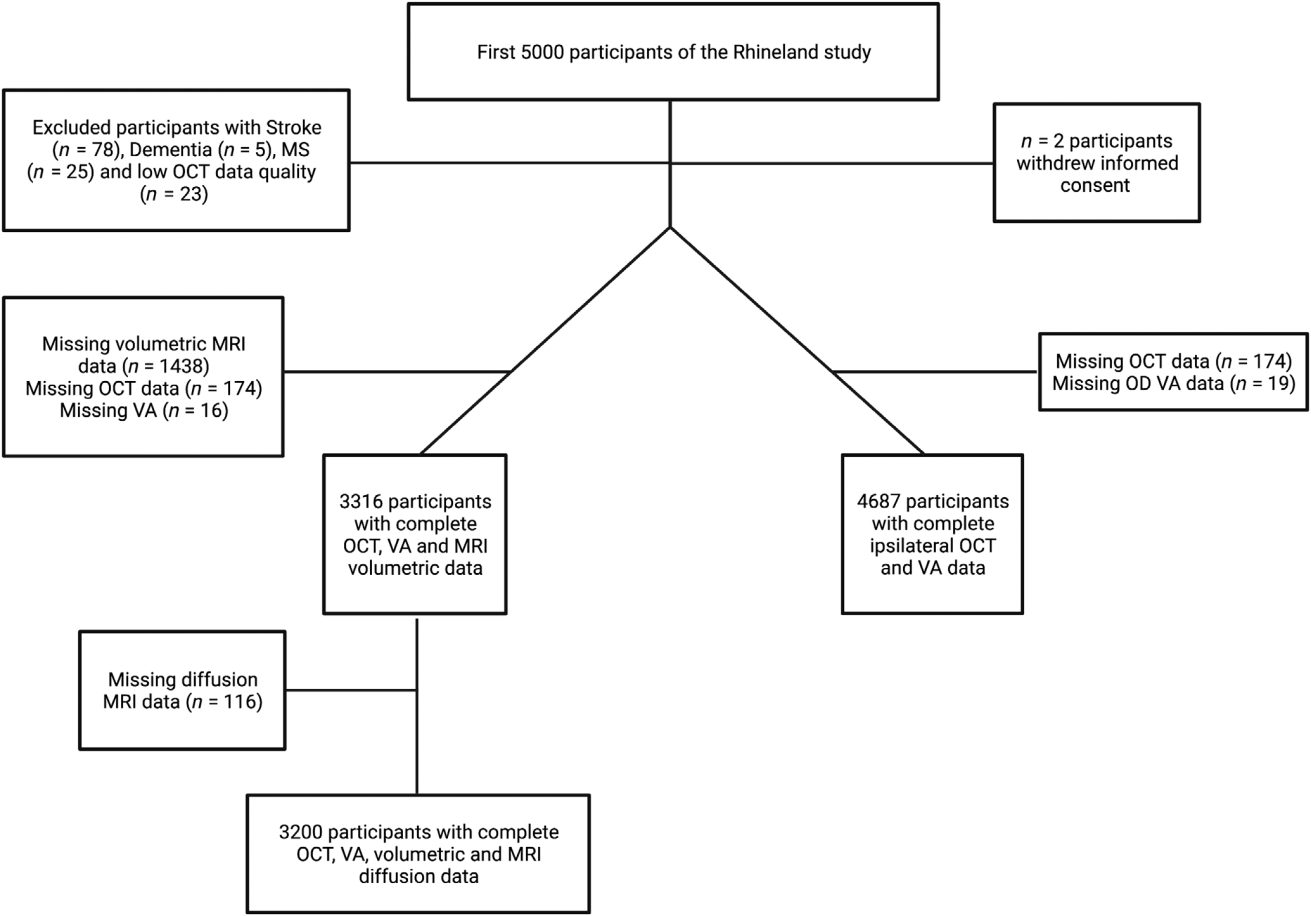
Flow chart of the study population. The most common reasons for missing volumetric brain magnetic resonance imaging (MRI) data were contraindications for MRI (*n* = 789, 52.8%), followed by refusal of brain imaging (*n* = 556, 37.2%), failed postprocessing (*n* = 5), and unknown reasons (*n* = 64). Further missings in diffusion MRI are due to postprocessing failure and drop‐out of the diffusion part of the MRI examination. The most common reasons for missing spectral domain (SD)‐optical coherence tomography (OCT) data were exclusion during quality assurance (*n* = 75, 43.1%), followed by technical issues (*n* = 72, 41.4%), and low participant compliance (*n* = 10, 5.7%). VA, visual acuity.

We analyzed baseline data from the first 5000 participants of the Rhineland Study, a community‐based prospective cohort study including inhabitants older than 30 years from two distinct regions in the city of Bonn, Germany (Mauschitz et al., 2019). We excluded participants with self‐reported neurological disorders (including stroke *N* = 78, dementia *N* = 5, and multiple sclerosis *N* = 25) and participants with insufficient SD‐OCT image quality (internal quality parameter ≤20 dB, *N* = 23; Figure 1). When assessing the relationship between ipsilateral (right eye) retinal volume and best‐corrected visual acuity (VA), 4687 participants were included (Table 2). The Rhineland study has been conducted according to the provisions of the Declaration of Helsinki, approved by the local ethic committee and all participants provided written informed consent.

### 2.2 Magnetic resonance imaging image acquisition

Magnetic resonance imaging (MRI) scans were acquired on 3 T Siemens MAGNETOM Prisma MRI scanners (Siemens Healthcare, Erlangen, Germany) equipped with 64‐channel head–neck coils at two examination sites in Bonn. The imaging protocol included a 3D T1‐weighted multiecho magnetization‐prepared rapid gradient‐echo sequence with 2D acceleration and elliptical sampling (acquisition time [TA] = 6.5 min; Brenner et al., 2014; 4 echo times between 1.7 and 6.5 ms, repetition time = 2560 ms, inversion time = 1100 ms, flip angle = 7°, field of view = 256 × 256 mm^2^, 224 slices, 0.8 mm isotropic resolution). Simultaneous multislice diffusion‐weighted MRI (dMRI) was performed with a spin‐echo echoplanar imaging sequence applying threefold slice acceleration (Setsompop et al., 2012). A compressed sensing diffusion spectrum imaging protocol was used to collect dMRI scans at 1.5 mm isotropic spatial resolution (Harms et al., 2017; Lohner et al., 2022). The MRI protocol also included 3D sequences to acquire T2‐weighted and FLAIR images (Lohner et al., 2022).

### 2.3 MRI image processing

**FIGURE 2:**
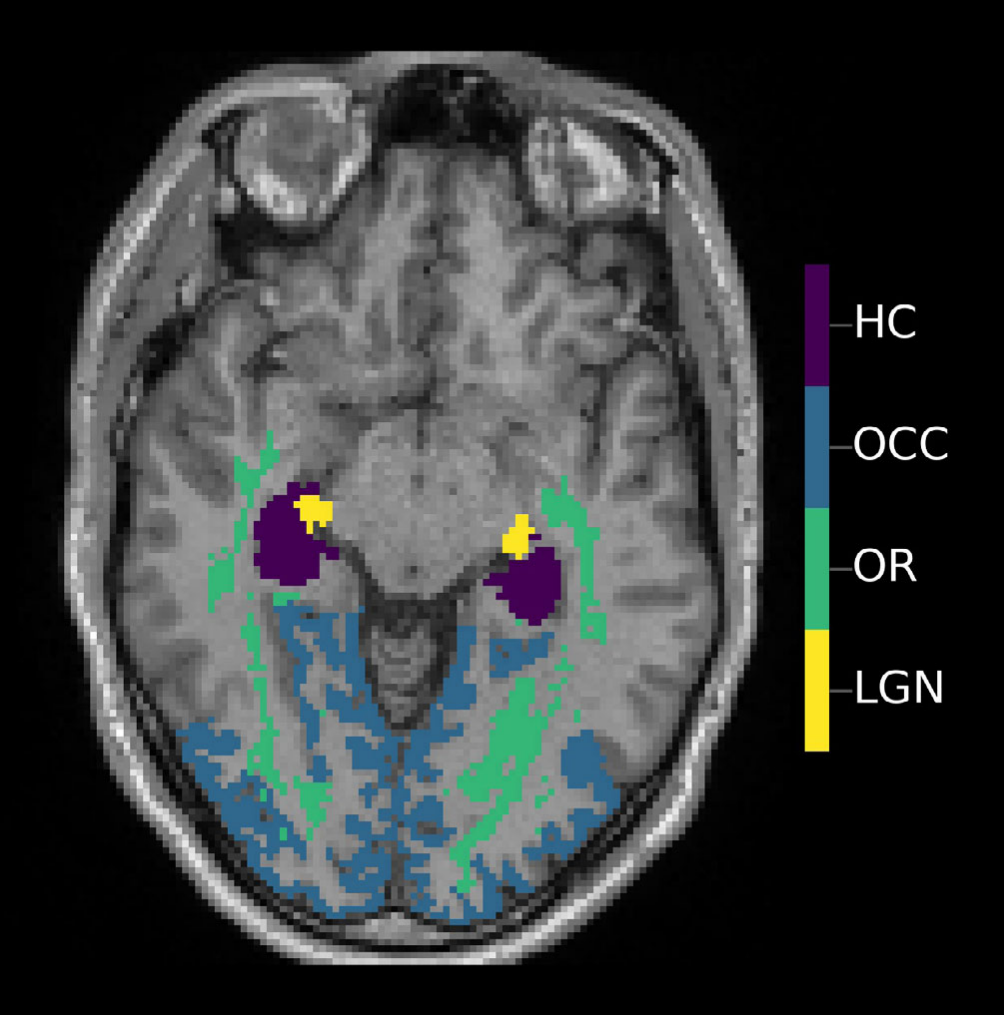
Axial view of brain regions for the lateral geniculate nucleus, optic radiation, pericalcarine cortex, and visual association areas obtained from diffusion magnetic resonance imaging and T1 FreeSurfer processing, and overlaid onto a T1‐weighted scan. HC, hippocampus; LGN, lateral geniculate nucleus; OCC, occipital lobe; OR, optic radiation.

All T1‐weighted images were processed using FreeSurfer version 6.0 (http://surfer.nmr.mgh.harvard.edu/) to derive quantitative volumetric measures (Fischl, 2012; Fischl et al., 2002). We used the estimated total intracranial volume generated by FreeSurfer as a proxy for head size (Buckner et al., 2004). Processing steps for diffusion MRI included the correction of susceptibility‐induced and eddy current‐induced distortions and head motion using FSL version 6.0 (www.fmrib.ox.ac.uk/fsl), compressed sensing reconstruction and subsequent estimation of fractional anisotropy (FA) from the diffusion tensor model through voxel‐wise model fitting using the Microstructure Diffusion Toolbox framework (Andersson et al., 2003; Basser et al., 1994; Harms et al., 2017; Tobisch et al., 2018, 2019). A whole brain white matter mask was obtained from the T1‐weighted MRI data using the standard FreeSurfer processing pipeline, corrected for white matter hyperintensities determined based on T1‐weighted, T2‐weighted, and FLAIR images, and further refined through FA skeletonization. Using the Jülich histological atlas, white matter tract‐specific and gray matter average FA values were derived for the optic radiation and the lateral geniculate nucleus, respectively (Figure 2; Bürgel et al., 2006).

### 2.4 Brain regions of interest

We included brain areas known to be involved in visuosensory and cognitive processing. As measures of visual pathway integrity, we assessed the FA of the lateral geniculate nucleus, the optic radiation and the occipital lobe. As cortical areas involved in ventral stream higher order visual processing, we selected the inferior temporal, fusiform, and parahippocampal cortex (Aminoff et al., 2013; Zilles & Amunts, 2012). We also included the volume of the hippocampus, because of its importance as a cognitive biomarker (Jack et al., 1998), as well as total brain volume as a measure of brain atrophy.

### 2.5 SD‐OCT image acquisition and processing

We assessed retinal layers using Spectralis SD‐OCT (Heidelberg Engineering, Heidelberg, Germany) as described previously (Mauschitz et al., 2019). In brief, the SD‐OCT imaging protocol includes a macular volume scan (97 horizontal B‐scans, 20 automatic real‐time [ART] frames per B‐scan, 20° × 20° field) and two circular OCT scans around the optic nerve head (3.5 mm diameter circular scan with 100 ART frames and 24 radial scans with 25 ART frames each). Volumes of six macular layers (RNFL, GCL, IPL, inner nuclear layer (INL), outer plexiform layer (OPL), outer nuclear layer (ONL), and retinal pigment epithelium (RPE)), total retinal volume, and peripapillary RNFL (pRNFL) thickness were computed using the inbuilt segmentation algorithm of the Heidelberg Eye Explorer (HEYEX). For each layer, we utilized total volume in mm^3^ except for the pRNFL, which is computed as average thickness in μm. Corneal curvature of individuals was entered before scan acquisition to adjust for corneal refraction.

### 2.6 Visual acuity and spherical equivalent

Refraction and VA were measured with an automated refractometer (Ark‐1s; Nidek Co., Tokyo, Japan). Values <0.1 were manually assessed with a Snellen chart. Qualitative measurements of low‐range VA (*n* = 44) were imputed as follows: perception of hand movements, finger counting, and light/nulla lux was set to 0.005, 0.014, and 0.001, respectively (Schulze‐Bonsel et al., 2006). Spherical equivalent was calculated as the spherical value and half of the cylindrical value. We utilized VA from the right eye when assessing the relationship with ipsilateral retinal measures (Table 2), and the average of the two eyes when assessing the relationship with brain measures, since each eye sends information to both ipsilateral and contralateral brain hemispheres. If VA was missing in one eye, we utilized VA from the contralateral eye. We utilized retinal measures from the right eye since it is the first examined eye in our imaging protocol, and therefore contains fewer missing values for retinal measurements.

### 2.7 Statistical analysis

Descriptive statistics are presented as means and standard deviation for continuous variables and as frequency and percentages for categorical variables. VA is presented as the logarithm of the minimum angle of resolution (LogMar scale), with higher values indicating worse vision. All brain imaging measures were averaged between the two hemispheres. The variable “Higher‐order visual areas” was a composite average of Z‐scores of selected brain areas (i.e., inferior, middle temporal, and fusiform gyrus). We used multivariable linear regression models to investigate which retinal layer had the strongest association with VA and total brain volume. Similarly, we used multivariable linear regression to assess the associations between VA, GCL volume and optic radiation, lateral geniculate nucleus, occipital lobe, higher order visual association areas, hippocampal, and total brain volume.

In all models, VA was included as an independent variable; retinal measures were included as dependent variables when regressed on VA, and as independent variables when assessing their effect on brain structure. Statistical significance was inferred at a two‐tailed, false discovery rate‐corrected *q* < 0.05.

Structural equation modelling was used for mediation analysis. To investigate whether retinal neurodegeneration mediates the effect of VA on brain atrophy and hippocampal volume, we included GCL volume as a mediator. To assess whether the effect of retinal neurodegeneration on brain atrophy and hippocampal volume is mediated by transneuronal degeneration along the visual pathway, we included FA measures of visual pathway (lateral geniculate nucleus, optic radiation) and occipital lobe volume as potential mediators. For structural equation modeling, we standardized continuous variables using Z‐scores to allow for estimate comparison. The 95% confidence intervals (CIs) of all the mediation analyses estimates were based on nonparametric bias‐corrected bootstrapping with 1000 resamplings.

**FIGURE 3:**
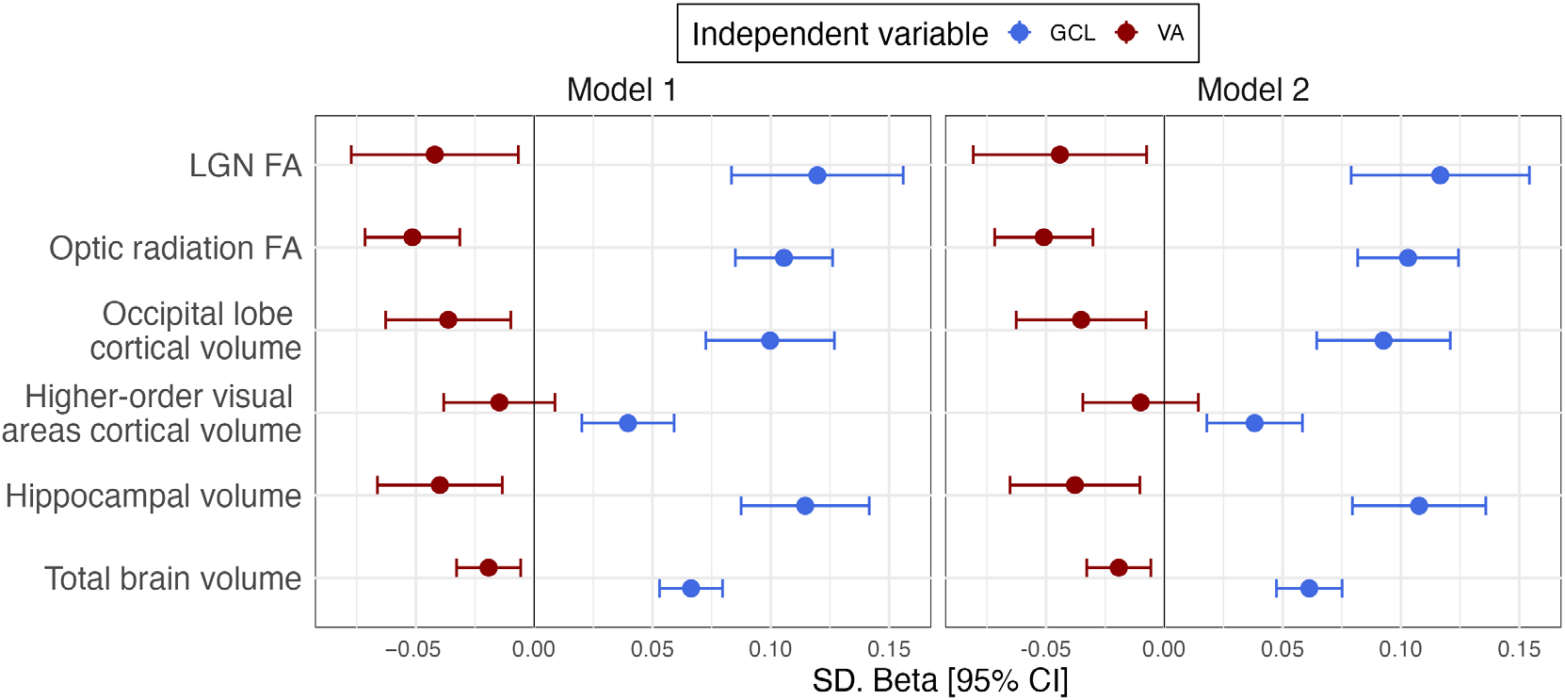
Associations between visual acuity, retinal volume, and cerebral measures. Each row represents one distinct regression model with dependent variables listed on the left side of the figure. Model 1 is adjusted for age, sex, intracranial volume for volumetric measures, global FA for optic radiation FA, SBP, and spherical equivalent for models including GCL. Model 2 is additionally adjusted for smoking status, diabetes mellitus, cholesterol levels, and body mass index. Visual acuity was quantified as the logarithm of the minimum angle of resolution with optimal correction of refractive errors. FA, fractional anisotropy; GCL, ganglion cell volume; LGN, lateral geniculate nucleus; VA, best‐corrected visual acuity. FDR *q* *q < 0.05, **q < 0.01, ***q ≤ 0.0001.

We adjusted all models, both in the regression analysis and in the structural equation modeling, for age, sex, systolic blood pressure, and spherical equivalent as a proxy of axial length when including retinal measures, and intracranial volume when considering brain volumetric measures (Mauschitz et al., 2019). We additionally adjusted the regression models for known cerebrovascular risk factors (including smoking status, diabetes mellitus, cholesterol levels, and body mass index) to ensure that the associations were independent of these cerebrovascular risk factors (Model 2 in Figure 3). Optic radiation FA was further adjusted for global brain FA, to assess the effect of visual pathway degeneration independently from whole brain pathology.

### 2.8 Sensitivity analyses

We assessed the robustness of our findings by excluding individuals with extreme values of VA, GCL, spherical equivalent, and self‐reported age‐related macular degeneration (AMD; *n* = 69) and glaucoma (*n* = 65). We also tested the robustness of our mediation analysis results by including models with “control” areas in the frontal lobe not in direct synaptic contact with visual pathway structures.

All statistical analyses were performed in R (base version 3.4), using the “lavaan” package for structural equation modeling (Rosseel, 2012).

## 3 RESULTS

### 3.1 Participant characteristics

An overview of the characteristics of the study participants is presented in Table 1.

**TABLE 1:** Characteristics of the study population.

	Analytical population	MRI or OCT or VA missing data	*p*‐Value
*N*	3316	1552	
Sex (male)a	1386 (41.8)	729 (47.0)	.001
Age (mean ± SD) (range)	54.49 ± 13.5 (30.2–95.4)	57.53 ± 14.6 (30.5–93.2)	<.001
Average VA from both eyes (mean ± SD)	−0.01 **±** 0.14	0.03 **±** 0.24	<.001
High (−0.1–0.1)^a^	2884 (87.0)	1244 (81.0)	
Middle (0.11–0.4)^a^	382 (11.5)	233 (15.1)	
Low (>0.41)^a^	50 (1.5)	59 (3.8)	
SBP (mean ± SD) (range)	125.9 ± 15.80	127.2 ± 16.46	.008
GCL volume (mean ± SD) mm^3^	1.07 ± 0.10	1.06 ± 0.11	.072
TRET volume (mean ± SD) mm^3^	8.65 ± 0.41	8.63 ± 0.41	.111
OR FA (mean ± SD)	0.63 ± 0.02	—	
TB volume (mean ± SD) mm^3^	1,109,230 (117,960)	—	

Abbreviations: GCL, ganglion cell volume; OR FA, optic radiation fractional anisotropy; SBP, systolic blood pressure; SD, standard deviation; TB, total brain; TRET, total retinal volume; VA, visual acuity.

^a^ Sex and strata of visual acuity are presented as counts (frequency as percentage). Two sample *t*‐tests were used for intergroup comparisons.

Participants with missing data were significantly more often male, older, and had worse average vision and higher blood pressure. The majority of participants 2884 (91.4%), had good VA (−0.1–0.1), while it was reduced or poor in 270 participants (8.6%). Among 158 (4.8%) individuals with VA >0.2 (<0.63 on Snellen scale): 34 (21.5%) had been graded with early stages of AMD and 3 (1.9%) with late AMD. In total, 11 (7%) reported a diagnosis of glaucoma, 2 (1.3%) reported a history of uveitis, 12 subjects (7.6%) were graded with clinically significant lens opacities and 8 (5%) with epiretinal gliosis. In 2 individuals, retinal vascular disease and signs of myopic degeneration were graded, respectively.

### 3.2 Association between VA, retinal layer measures, and total brain volume

All retinal layer measures were significantly associated with both VA and total brain volume, except the INL and the RPE, which were only associated with total brain volume. Standardized effect sizes, allowing for comparison of the strength of the associations between different retinal layers, are reported in Table 2. One‐unit worse VA was associated with a 0.06 mm^3^ thinner GCL [95% CI −0.08 to −0.04], while a 0.1 mm^3^ decrease in GCL was associated with a 7524 mm^3^ decrease in total brain volume [95% CI 6013–9035]. Among all inner retinal layer measures, VA had the largest effect on GCL, which in turn was most robustly associated with total brain volume. Other inner retinal layers showed similar trends, except for the OPL: one‐unit better VA (decrease on LogMar scale) corresponded to a 0.046 mm^3^ increase in OPL volume. Although a similar trend was discernible for the RPE, the association between its volume and VA did not reach statistical significance.

**TABLE 2:** Association between ipsilateral retinal measures, visual acuity, and total brain volume.

Retinal layer	Visual acuity^a^	Total brain volume^a^
pRNFL	−0.065 *** (−0.095 to −0.035)	0.047 *** (0.034–0.059)
GCL	−0.090 *** (−0.118 to −0.062)	0.066 *** (0.053–0.080)
IPL	−0.058 ** (−0.086 to −0.029)	0.062 *** (0.049–0.075)
INL	−0.004 (−0.033 to 0.026)	0.019 * (0.006–0.032)
OPL	0.095 *** (0.064–0.126)	0.018 * (0.005–0.030)
ONL	−0.055 ** (−0.085 to −0.025)	0.014 * (0.001–0.027)
RPE	0.026 (−0.005 to 0.058)	0.026 *** (0.014–0.038)
TRET	−0.041 * (−0.070 to −0.012)	0.050 *** (0.037–0.062)

*Note*: Each row represents one distinct regression model. Retinal measures were included as dependent variables when regressed on VA and as independent variables when assessing their effect on brain structure.

Abbreviations: FDR, false discovery rate; GCL, ganglion cell layer; INL, inner nuclear layer; IPL, inner plexiform layer; ONL, outer nuclear layer; OPL, outer plexiform layer; pRNFL, peripapillary nerve fiber layer; RPE, retinal pigmented epithelium; TRET, total retinal volume.

^a^ Visual acuity was quantified as the logarithm of the minimum angle of resolution corrected for refractive error (LogMar scale), with higher values representing worse vision. All multivariable linear regression models are adjusted for age, sex, systolic blood pressure, spherical equivalent and, when including brain volume, also for intracranial volume. FDR *q < 0.05; **q < 0.001 ; ***q < 0.0001.

The trend of the association between all retinal layers and brain volume was positive (Table 2). The association between VA and GCL became nonsignificant after excluding participants in the smallest decile of GCL volume (E‐Table 6), but remained robust in other sensitivity analyses, indicating that the effect of VA on GCL is likely driven by volumes in the lowest range.

### 3.3 Associations among the different components of the visual system

**FIGURE 4:**
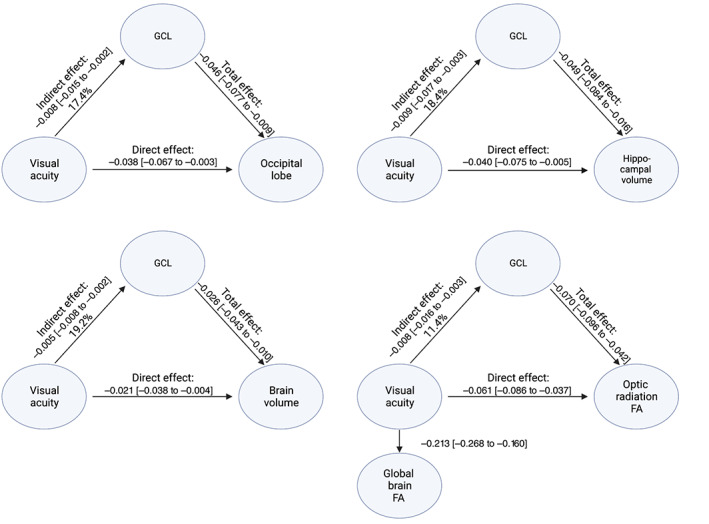
Inner retinal thinning mediates the effect of visual acuity on brain structural measures. All models are adjusted for age, sex, intracranial volume for volumetric measures, global FA for optic radiation FA, SBP, and spherical equivalent for models including GCL. Full lines indicate nominally significant effects, dashed lines are non‐nominally significant effects. Visual acuity was quantified as the logarithm of the minimum angle of resolution at optimal correction of refractive errors. FA, fractional anisotropy; GCL, Ganglion cell layer; SBP, systolic blood pressure.

Lower VA was associated with decreased occipital lobe, hippocampal, and total brain volume, as well as decreased FA of the optic radiation and the lateral geniculate nucleus (Figure 3). An association between VA and higher order visual areas was, however, not observed. GCL volume was strongly associated with all selected structural and FA measures of the visual pathway, including higher order visual association areas and hippocampus. Effect estimates changed little to none after additional adjustment (Model 2, Figure 3), indicating that these associations are independent from common cerebrovascular risk factors. Interestingly, VA also had a large effect on global FA, independent of its effect on optic radiation FA (Figure 4). Associations of VA and GCL with individual brain areas are reported in E‐Table 1. Briefly, the association between VA and occipital lobe structures was most pronounced in the lateral occipital and lingual lobe, while GCL was robustly associated with selected brain areas involved in processing of visual information. Importantly, the observed associations remained robust in all sensitivity analyses (E‐Tables 5, 7, and 8).

### 3.4 GCL volume mediates the effect of visual acuity on brain measures

GCL volume significantly mediated the association between VA and structures of the visual pathway (percentages of mediated effect of 17.4% and 11.4% for occipital lobe and optic radiation FA, respectively; Figure 4). The percentage of mediated effect increased to 18.4% and 19.2% for hippocampal and total brain volume, respectively. Interestingly, we observed a strong association between VA and global FA (1 SD decrease in VA corresponded to 0.21 SD decrease in global brain FA 95% CI 0.16–0.27; Figure 4).

### 3.5 Visual pathway structures mediate the effect of GCL on total brain and hippocampal volume

**FIGURE 5:**
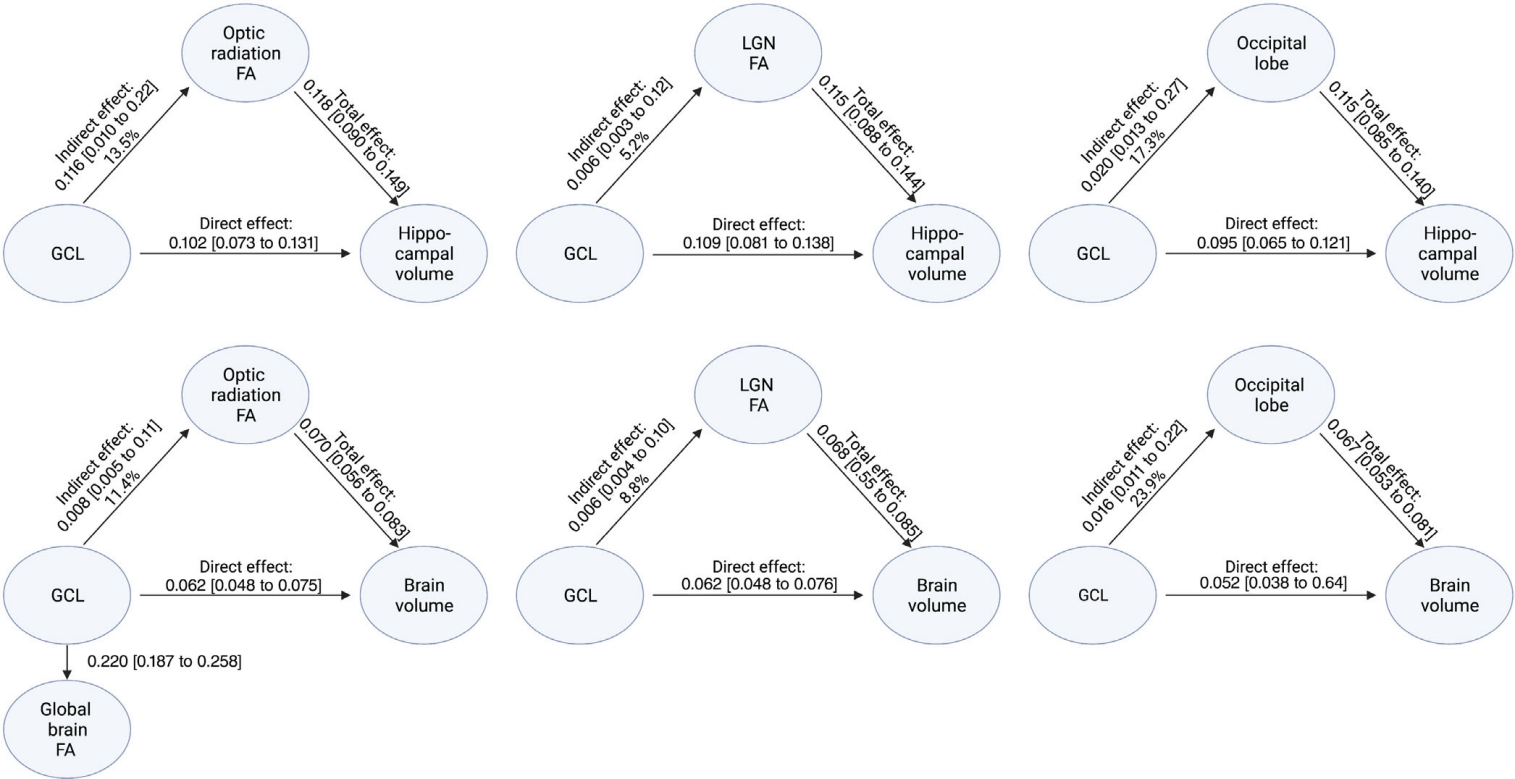
The effect of ganglion cell layer degeneration on total and hippocampal atrophy is mediated through disintegration of intermediate visual pathway tracts. All models are adjusted for age, sex, intracranial volume for volumetric measures, global FA for models including optic radiation FA, SBP, and spherical equivalent for models including GCL. All models are nominally significant. FA, fractional anisotropy; GCL, ganglion cell layer; SBP, systolic blood pressure.

The effect of GCL volume on total brain and hippocampal volume was mediated by all key structures of the visual pathway, including the lateral geniculate nucleus, the optic radiation and the occipital lobe (Figure 5). We observed the largest mediation effect sizes for the occipital lobe, accounting for 17.3% and 23.9% of the total effect for hippocampal and total brain volume, respectively. Importantly, apart from its relation to the optic radiation, smaller GCL volume was also independently associated with lower global brain FA (i.e., 1 SD increase in GCL volume corresponded to 0.22 SD increase in global brain FA 95% CI 0.18–0.26; Figure 5).

In the sensitivity analyses, we did not find an association of VA with two “control” brain areas in the frontal lobe not in direct synaptic contact with visual pathway structures. In the structural equation models, we observed a weaker association between GCL and the middle frontal lobe volume, which was not mediated by optic radiation and lateral geniculate nucleus FA (E‐Table 3). The occipital lobe volume fully mediated the effect of GCL on the middle frontal lobe volume (E‐Table 4).

## 4 INTERPRETATION

We present a large‐scale population‐based study in which we systematically assessed visual impairment in relation to the structural integrity of key components of the central visual system, including all retinal layers, visual pathway microstructure, as well as higher order cortical and subcortical visual association areas. We found that GCL is the inner neuronal retinal layer most robustly associated with both visual impairment and brain atrophy. This association was mainly due to individuals very low GCL volume, but was robust to exclusion of individuals with ophthalmological diseases, low VA, and large refractive errors. Interestingly, VA was also robustly associated with larger OPL; however, the direction of this association was opposite to that for inner retinal layers. Moreover, we observed that visual impairment was associated with decreased structural integrity, measured as lower FA, of all major components of the visual pathway, as well as with lower total brain and hippocampal volume. VA had a much larger effect on optic radiation and global FA than on brain volumetric measures, indicating that microstructural changes might be more sensitive to visual impairment than atrophy. In additional sensitivity analyses, we observed that our findings were robust to exclusion of very low values of VA (LogMar 0.4), extreme refractive errors, and common ophthalmological conditions such as glaucoma and AMD, indicating that subclinical brain changes may occur at milder levels of visual impairment and might be a consequence of any visual impairment regardless of its cause. Importantly, conjoint modeling of the neuroanatomical connections between visual impairment and the different components of the central visual system indicated a pattern consistent with transneuronal degeneration. Thus, our findings imply that visual impairment and degeneration of retinal and central visual pathway structures are closely related, with pathology of any of these components likely to propagate along the entire chain through transneuronal degeneration.

Our findings are in line with previous studies reporting an association between visual impairment and transneuronal degeneration along the visual pathway (Boucard et al., 2009; Hanson et al., 2019; Hernowo et al., 2011, 2014; Malania et al., 2017). Our finding of an association between visual impairment and both total and hippocampal atrophy corroborates previous reports of a link between visual and cognitive impairment (Chen et al., 2017; Lee et al., 2021). We also observed that retinal neurodegeneration partly mediates the association between visual impairment and brain structural integrity. Although the association between visual impairment and retinal neurodegeneration is likely to be bidirectional, we specifically assessed mediation through GCL volume, which can be interpreted as how visual impairment could affect brain structure independently of (causes related to) inner retinal thinning, such as transneuronal neurodegeneration. Indeed, retinal neurodegeneration was associated with decreased structural integrity measures across the entire visual pathway, which also partly mediated the association between retinal neurodegeneration and both total brain and hippocampal atrophy. Our findings support the notion that retinal neurodegeneration, in part reflecting visual impairment, may reflect changes in the visual pathway up to and including the occipital lobe, leading to decreased activation and degeneration of other interconnected brain regions, ultimately resulting in cerebral atrophy and cognitive decline.

Several studies describing retinal changes in neurological diseases such as Alzheimer disease and multiple sclerosis, have hypothesized involvement of the visual system in the pathophysiology of these disorders (Chan et al., 2018; Mckee et al., 2006; Mirzaei et al., 2020). For example, in Alzheimer disease, visual areas are a primary site of amyloid deposition, while visuospatial symptoms even predominate in the posterior cortical atrophy variant of the disease (Mckee et al., 2006; Mirzaei et al., 2020). Moreover, retinal and brain amyloid deposition might be correlated, with the highest correlation seen in the pericalcarine lobe (Koronyo et al., 2017). Our observation that the direct effect between retinal neurodegeneration and brain atrophy was consistently larger in magnitude than the indirect effect through visual pathway structures, suggests that apart from transneuronal degeneration, other (shared) etiopathological mechanisms may also account for part of the association. Neuronal damage, due to aging or other pathologies, might lead to neuronal death which is reflected in degeneration of both retinal and cerebral structures. Retinal neurodegeneration therefore also likely reflects central neurodegeneration, independently of visual pathway decay (Garzone et al., 2022). Studies investigating retinal changes in brain diseases with predominant visual involvement and including more granular measures of visual function could further elucidate this relationship.

Indeed, a general neurodegenerative component may also explain the relatively weak direct effect of retinal neurodegeneration on one included “control” area (i.e., middle frontal lobe), although this effect was smaller in magnitude and less robust than for visual pathway areas.

Interestingly, a recent study found increased hippocampal amyloid clearance after exposing Alzheimer's disease transgenic mice to noninvasive light flickering at specific frequencies, thought to be due to restoration of local circuits of excitatory and inhibitory neurons (Iaccarino et al., 2016). Visual stimulation in patients with neurodegenerative diseases may also enhance the release of neurotrophic factors and thereby improve neuronal resilience and plasticity (Yang et al., 2021). In line with these findings, a recent study found a reduction in dementia risk after visual restoration through cataract surgery (Lee et al., 2021), while previous studies observed increased dementia risk in presence of ophthalmic conditions (Klaver & De Jong, 1999; Shang et al., 2021). Our results extend these previous findings by showing that in the general population, even relatively mild degrees of visual impairment and retinal degeneration are robustly associated with worse imaging indices of brain integrity, partly mediated through transneuronal degeneration. Therefore, our findings suggest that treatment of even mild degrees of visual impairment and retinal pathology might help warding off (age‐associated) neurodegeneration and cognitive decline.

Our study has both strengths and limitations. Strengths of our study include the large sample size covering a wide age range, a relatively homogenous population‐based sample, the employment of detailed imaging markers of both retinal layers and brain structure and connectivity, and concomitant assessment of visual impairment in relation to all key components of the central visual system, allowing for in‐depth modelling of their neuroanatomical interconnections. Potential limitations of our study include the lack of more detailed measures of visual function, as VA was approximated using a per chart row and not per optotype. It has been observed that transsynaptic neurodegeneration in the visual pathway proceeds at a certain rate, implying different cerebral effects between early‐onset and late‐onset visual impairment (Dinkin, 2017; Jindahra et al., 2012). Unfortunately, due to lack of relevant data, we could not analyze the effects of onset and duration of visual impairment on brain integrity measures. Additionally, we accounted for common vascular risk factors that are associated with visual impairment, retinal, and brain changes in our analyses: we excluded individuals with stroke or large cerebral vascular lesions on MRI, adjusted our analyses for several cardiovascular risk factors, and only considered normally appearing white matter for DTI measures; however, some residual confounding might persist through small brain vascular lesions. Furthermore, to aid interpretability of our findings we modeled visual acuity as the average of the two eyes to capture decreased visual brain input, which is considered a valid approach when investigating outcomes operating at an individual level, such as brain areas atrophy. Although also more advanced and complex statistical methods of modeling information from the two eyes have been adopted and described before, the results are generally in line with the approach we have adopted (Murdoch et al., 1998; Ying et al., 2017). Finally, due to the population‐based design of the study our disease assessment was based on self‐reports, which may be subject to recall bias.

In conclusion, we found that both visual impairment and retinal neurodegeneration were associated with global as well as regional brain atrophy, partly mediated through disintegration of central visual pathways. Our findings thus not only support the use of OCT‐derived retinal measures as markers of neurodegeneration, but also indicate that treatment of visual impairment and retinal pathology may be easily actionable, inexpensive measures for the maintenance of brain health and prevention of (age‐associated) neurodegeneration and cognitive decline.

## AUTHOR CONTRIBUTIONS

**Davide Garzone:** conception and design of the study, acquisition and analysis of data, and drafting the text/figures. **Robert P. Finger:** conception and design of the study, acquisition of data, and reviewing the text/figures. **Matthias M. Mauschitz:** acquisition of data and reviewing the text/figures. **Alexandra Koch:** acquisition and analysis of data; drafting and reviewing the text/figures. **Martin Reuter:** acquisition of data and reviewing the text/figures. **Monique M. B. Breteler:** conception and design of the study, acquisition of data, and reviewing the text/figures. **N. Ahmad Aziz:** conception and design of the study, acquisition of data; reviewing the text/figures.

## CONFLICT OF INTEREST STATEMENT

The author Robert P. Finger declares grants and personal fees from Novartis; grants from Biogen; personal fees from Bayer, Santen, Ophtea, Apellis, Roche/Genentech, Böhringer‐Ingelheim, Novelion, ProQR, Oxford Innovation, Roche, Alimera, Santhera, Inositec, and Ellex. Other authors declare no conflict of interest.

## Supporting information


**Data S1:** Supporting information.

## Data Availability

The datasets for this article are not publicly available because of data protection regulations. Access to data can be provided to scientists in accordance with the Rhineland Study's Data Use and Access Policy. Requests for further information or to access the datasets should be directed to RS‐DUAC@dzne.de.
