# Supplementary material for: Visual impairment and retinal and brain neurodegeneration: A population‐based study
Source: Hum Brain Mapp. 2023 Feb 28;44(7):2701–11. doi: 10.1002/hbm.26237 (PMC10089094; doi:10.1002/hbm.26237)
Supplement: Supplementary file 1 — Data S1: Supporting information. [file HBM-44-2701-s001.docx]

|  | Pericalcarine | Cuneus | Lingual | Lateral | Parahippocampal | Inferotemporal | Fusiform |
| --- | --- | --- | --- | --- | --- | --- | --- |
| VA  Model 1 | -0.018  (-0.051 – 0.016) | -0.029  (-0.058 – 0.001) | -0.039 * (-0.069 – -0.010) | -0.032 * (-0.059 – -0.005) | -0.004  (-0.036 – 0.028) | -0.007  (-0.031 – 0.018) | -0.024  (-0.049 – -0.000) |
| VA Model 2 | -0.014  (-0.049 – 0.021) | -0.028  (-0.059 – 0.003) | -0.036 * (-0.067 – -0.005) | -0.033 * (-0.061 – -0.005) | 0.002  (-0.032 – 0.035) | -0.005  (-0.031 – 0.021) | -0.020  (-0.046 – 0.005) |
| GCL  Model 1 | 0.136 *** (0.101 – 0.170) | 0.080 *** (0.049 – 0.110) | 0.108 *** (0.078 – 0.139) | 0.065 *** (0.037 – 0.092) | 0.050 * (0.017 – 0.083) | 0.035 * (0.010 – 0.061) | 0.032 * (0.007 – 0.057) |
| GCL Model 2 | 0.127 *** (0.091 – 0.164) | 0.076 *** (0.044 – 0.108) | 0.103 *** (0.071 – 0.135) | 0.057 ** (0.029 – 0.086) | 0.048 * (0.014 – 0.083) | 0.032 * (0.006 – 0.059) | 0.032 * (0.006 – 0.058) |

**E-Table 1. Associations with individual brain areas**

Legend: VA = best-corrected visual acuity, GCL =Ganglion cell layer.

FDR q* <0.05, ** q<0.01, *** q<=0.0001

Model 1 is adjusted for age, sex, intracranial volume, spherical equivalent for models including GCL and SBP. Model 2 is additionally adjusted for smoking status, diabetes mellitus, cholesterol levels and body-mass index.

| Predictor | Mediator | Outcome | Root Mean Square Error of Approximation (95%CI) |
| --- | --- | --- | --- |
| VA | GCL | Occipital lobe | 0.055 (0.035 – 0.077) |
| VA | GCL | Total brain volume | 0.054 (0.035 – 0.076) |
| VA | GCL | OR FA | 0.136 (0.124 – 0.128) |
| VA | GCL | Hippocampal volume | 0.054 (0.035 – 0.076) |
| GCL | OR FA | Hippocampal volume | 0.146 (0.105 – 0.127) |
| GCL | LGN FA | Hippocampal volume | 0.140 (0.120 – 0.162) |
| GCL | Occipital lobe | Hippocampal volume | 0.000 (0.00 – 0.045) |
| GCL | LGN FA | Total brain volume | 0.140 (0.120 – 0.162) |
| GCL | OR FA | Total brain volume | 0.146 (0.135 – 0.158) |
| GCL | Occipital lobe | Total brain volume | 0.000 (0.00 – 0.00) |

**E-Table 2. Measures of fit for structural equation models**

# Sensitivity analysis 1, “Control” brain areas

| **Outcome** | **Visual acuity** | **GCL** |
| --- | --- | --- |
| **Middle frontal lobe** | -0.0093 [-0.0416 – 0.0229] | 0.0326 [0.0058 – 0.0594] * |
| **Frontal pole** | -0.0019 [-0.0382 – 0.0422] | 0.0065 [-0.0260 – 0.0392] |

**E-Table 3**, Regression analysis adjusted for age, sex, systolic blood pressure and spherical equivalent with “control” areas in the frontal lobe as outcomes, not in direct synaptic connection with the retina and visual pathway.

| GCL 🡪 OR FA 🡪 Middle frontal lobe | | | GCL 🡪 Occipital lobe 🡪 Middle frontal lobe | | |
| --- | --- | --- | --- | --- | --- |
|  | Beta | 95% CI |  | Beta | 95% CI |
| indirect | 0.000 | [-0.006 – 0.006] | indirect | 0.021 | [0.015 – 0.030] |
| direct | 0.005 | [-0.028 – 0.041] | direct | 0.008 | [-0.017 – 0.036] |
| total | 0.006 | [-0.029 – 0.038] | total | 0.029 | [0.002 – 0.057] |
| GCL 🡪 LGN FA 🡪 Middle frontal lobe | | | GCL 🡪 OR FA 🡪 Frontal pole | | |
|  | Beta | 95% CI |  | Beta | 95% CI |
| indirect | -0.003 | [-0.006 – 0.000] | indirect | 0.000 | [-0.006 – 0.006] |
| direct | 0.035 | [0.009 – 0.065] | direct | 0.005 | [-0.028 – 0.041] |
| total | 0.032 | [0.006 – 0.062] | total | 0.006 | [-0.029 – 0.038] |
| GCL 🡪 LGN FA 🡪 Frontal pole | | | GCL 🡪 Occipital lobe 🡪 Frontal pole | | |
|  | Beta | 95% CI |  | Beta | 95% CI |
| indirect | -0.005 | [-0.009 – 0.002] | indirect | 0.023 | [0.017 – 0.032] |
| direct | 0.011 | [-0.025 – 0.043] | direct | -0.023 | [-0.056 – 0.009] |
| total | 0.006 | [-0.029 – 0.039] | total | 0.000 | [-0.034 – 0.031] |

**E-Table 4**, Structural equation modeling including negative control areas in no direct synaptic connection with visual pathway areas in the frontal lobe as outcomes, adjusted for age, sex, systolic blood pressure and spherical equivalent.

# Sensitivity analysis 2, association between visual acuity and brain volume

|  | Model 1, VA | Model 2, VA |
| --- | --- | --- |
| Brain volume | -0.022 ** (-0.036 – -0.009) | -0.019 * (-0.033 – -0.005) |
|  | Model 3, VA | Model 4, VA |
| Brain volume | -0.032 *** (-0.045 – -0.019) | -0.027 *** (-0.040 – -0.014) |

**E-Table 5,** Effect of average visual acuity on brain volume after adjusting for age, sex, intracranial volume and SBP (Model 1) and adjusting additionally for DM, Cholesterol, BMI and smoking (Model 2), upon exclusion of extreme values of low visual acuity (>0.4 LogMar); after excluding individuals with graded intermediate and late AMD (n=174, Model 3) and subjects with self-reported ophthalmological diseases (Glaucoma n=62, AMD n=69, Model 4).

FDR q* <0.05, ** q<0.01, *** q<=0.0001

# Sensitivity analysis 3, association between visual acuity and retinal volume

| Outcome | Model 1, VA | Model 2, VA | Model 3, VA |
| --- | --- | --- | --- |
| GCL | -0.056 ** (-0.085 – -0.028) | -0.022 (-0.053 – 0.008) | -0.078 *** (-0.107 – -0.049) |
|  | Model 4, VA | Model 5, VA |  |
|  | -0.089 *** (-0.118 – -0.060) | -0.085 *** (-0.113 – -0.057) |  |
| OPL | Model 1,VA | Model 2, VA | Model 3, VA |
|  | 0.107 *** [0.075 – 0.139] | 0.067 ** [0.034 – 0.100] | 0.081 *** [0.049 – 0.114] |
|  | Model 4, VA | Model 5, VA |  |
|  | 0.101 *** [0.069 – 0.133] | 0.090 *** [0.059 – 0.122] |  |

**E-Table 6**, Effect of average visual acuity on retinal measures after sensitivity analyses excluding values of visual acuity larger than 0.4 (Model 1), extreme values of GCL or OPL (Model 2, largest and smallest 5%), extreme values of spherical equivalent (Model 3, less than -6D and more than +3.5 Diopters), subjects with self-reported ophthalmological diseases (Glaucoma n=62, AMD n=69, Model 4) and graded intermediate and late AMD (n=174, Model 5).

FDR q* <0.05, ** q<0.01, *** q<=0.0001

# Sensitivity analysis 4, association between visual acuity and other brain regions

| OR FA | Model 1, VA | Model 2, VA |
| --- | --- | --- |
|  | -0.094 *** [-0.130 – -0.059] | -0.096 *** [-0.130 – -0.062] |
| LGN FA | Model 1, VA | Model 2, VA |
|  | -0.050 * [-0.086 – -0.013] | -0.055 ** [-0.090 – -0.020] |
| Hippocampal volume | Model 1, VA | Model 2, VA |
|  | -0.064 *** [-0.092 – -0.036] | -0.046 ** [-0.073 – -0.020] |
| Occipital lobe volume | Model 1, VA | Model 2, VA |
|  | -0.049 ** [-0.077 – -0.021) | -0.032 * [-0.059 – -0.005] |

**E-Table 7**, Effect of VA on selected brain measures after sensitivity analyses eliminating VA less than 1 (Model 1) , self-reported ophthalmological diseases (Glaucoma n=62, AMD n=69, Model 2).

FDR q* <0.05, ** q<0.01, *** q<=0.0001

# Sensitivity analysis 5, association between retinal measures and brain volume

| Independent  variable | Model 1 | Model 2 | Model 3 |
| --- | --- | --- | --- |
| GCL | 0.065 *** [0.051 – 0.079] | 0.069 *** [0.056 – 0.083] | 0.066 *** [0.053 – 0.080] |
| RPE | 0.020 * (0.007 – 0.033) | 0.025 ** (0.013 – 0.038) | 0.028 *** (0.015 – 0.040) |

**E-Table 8**, Effect of retinal measures on total after sensitivity analyses eliminating 5% largest and smallest retinal measure values (Model 1), self-reported ophthalmological diseases (Glaucoma n=62, AMD n=69, Model 2) and graded intermediate and late AMD (n=174, Model 5).

FDR q* <0.05, ** q<0.01, *** q<=0.0001
